# Evaluation of dermoscopic findings in patients with cutaneous squamous cell carcinoma according to histopathological subtype and lesion morphology^☆^

**DOI:** 10.1016/j.abd.2024.09.005

**Published:** 2025-02-13

**Authors:** Mustafa Ürün, Yıldız Gürsel Ürün, Ömer Faruk Elmas, Nuray Can

**Affiliations:** a Department of Dermatology, Faculty of Medicine, Trakya University, Edirne, Turkey; b Department of Dermatology, Medicana International Istanbul Hospital, İstanbul, Turkey; c Department of Pathology, Faculty of Medicine, Trakya University, Edirne, Turkey

**Keywords:** Bowen's disease, Carcinoma, squamous cell, Dermoscopy, Keratoacanthoma

## Abstract

**Background:**

Cutaneous squamous cell carcinoma (cSCC) includes in situ cSCC (Bowen's disease [BD]) and invasive cSCC. By contrast, keratoacanthoma (KA) is a well-differentiated cSCC with self-resolving tendencies. Dermoscopy aids in monitoring vascular and keratin pattern changes to diagnose and track cSCC invasion.

**Objectives:**

To examine dermoscopic findings of cSCC according to histopathological differentiation and clinical morphological characteristics.

**Methods:**

Clinical and dermoscopic images of 118 cSCCs were retrospectively examined.

**Results:**

Compared to other cSCC subtypes, BD more frequently presented with pigmentation (p = 0.028) and a clustered (p = 0.042) or serpiginous (p = 0.006) vascular arrangement. Central keratin plugs were more common in well-differentiated invasive cSCCs (p = 0.021), while white circles surrounding follicles (p < 0.001), ulceration/bleeding (p = 0.001), and red background (p = 0.004) were observed more in poorly differentiated invasive cSCCs. Central keratin plugs (87.5%) and branched vascular arrangements (75%) were observed in patients with nodular KA, with both statistically more frequent than in invasive cSCC (p < 0.001, p = 0.040, respectively). White halos surrounding vessels (p = 0.004) and a clustered vessel arrangement (p = 0.037) were more common in nodular invasive cSCC compared to nodular KA.

**Study limitations:**

The number of examined lesions in the subgroups was relatively small.

**Conclusions:**

Dermoscopy aids in distinguishing well-differentiated invasive cSCC from poorly differentiated invasive cSCC, distinguishing nodular KA from nodular invasive cSCC, and diagnosing BD. Further studies are needed to identify dermoscopic findings that can distinguish moderately differentiated invasive cSCC from other invasive cSCCs.

## Introduction

Cutaneous squamous cell carcinoma (cSCC) accounts for 20%‒30% of non-melanoma skin malignancies.^1^ cSCC encompasses a broad spectrum of clinical entities that vary in terms of cellular atypia and dysplasia and range from precursor lesions to *in situ* cSCC and invasive cSCC.^2^ There are three main subtypes of cSCC: Bowen’s disease (BD), keratoacanthoma (KA), and invasive cSCC.^3^

Dermoscopic findings of BD (i.e., *in situ* SCC) include a pink or pigmented background accompanied by clustered glomerular vessels and dotted vessels, yellow-white squams, and erosion.^4^ KA shares common clinical and histopathological features with well-differentiated cSCC.^5^ KA very rarely transforms to invasive cSCC and thus its accurate diagnosis prevents overtreatment.^6^

Invasive cSCC has specific dermoscopic signs related to its differentiation and depth of invasion.^7^ The dermoscopic structures seen in cSCC can be summarized as follows: 1) Keratin/central keratin, 2) Vascular structures, 3) Ulceration, and 4) White structureless areas.^8^ As invasive cSCC differentiates further, the keratin structures disappear, a polymorphic pattern of linear vessels covers the lesion, and ulceration develops.^9^ Histopathological differentiation is an important risk factor in cSCC tumor aggressiveness and recurrence risk.^3^, ^10^ Early differential diagnosis of the variants of cSCC is especially important considering the metastatic potential of invasive cSCC.^11^

This present study aimed to examine the distribution of dermoscopic findings in cases of invasive cSCC with different histopathological differentiation, as well as compare dermoscopic findings according to different morphological characteristics in cSCC patients and evaluate the dermoscopic findings of patients presenting with nodular invasive cSCC compared to those of patients with KA. The authors also aimed to further contribute to the literature by reviewing the dermoscopic findings of BD.

## Methods

### Statement of ethics

The study was approved by the Non-interventional Clinical Research Ethics Committee of the Trakya University Faculty of Medicine (2023/479).

### Patients

This retrospective cross-sectional study examined the records of 118 patients from January 2019 to July 2024 at the Trakya University dermatological and venereal diseases outpatient clinic. The study included patients who had a histopathological diagnosis of invasive cSCC, BD, or KA following complete surgical excision of the tumor, who had high-quality macroscopic and dermoscopic photographs, and who had sociodemographic and clinical data available”.

The patients’ sociodemographic characteristics (age, gender, past history of SCC, Fitzpatrick phototype), clinical characteristics (presence of multiple actinic keratoses at diagnosis, lesion duration, lesion location, whether in sun-exposed or sun-protected area),^12^ and histopathological diagnosis (well-differentiated cSCC, moderately differentiated cSCC, poorly differentiated cSCC, BD, or KA) were recorded by a member of the research team (M.Ü). The same researcher evaluated macroscopic images for lesion morphology (erythematous papule/plaque, hyperkeratotic papule/plaque, hyperkeratotic nodule, tumor, ulcerated papule/plaque, and ulcerated nodule) and classified the lesions as flat or elevated after evaluating their clinical features. Macroscopic photographs were collected from the clinic's image dataset. Patients who had invasive cSCC and KA with nodular morphology were evaluated in separate subgroups. As ulceration is morphologically rare in KA, ulcerated nodular lesions were not evaluated. The histopathological classification has been revised according to the diagnostic criteria set by the WHO in 2024 (N.C).^13^

### Dermoscopy

Dermoscopic images were obtained using 20× lenses and a dermoscopy system based on the FotoFinder platform (FotoFinder Systems GmbH, Germany). All images were obtained by contact dermoscopy with alcohol gel or ultrasound gel in patients with highly elevated lesions. Evaluation of dermoscopic images was performed independently by two researchers (Y.G.Ü & Ö.F.E) who were both blinded to the patient’s clinical information and histopathological diagnoses. Dermoscopic images were retrieved from the database of the digital video dermoscopy system at the clinic.

**Fig. 1:**
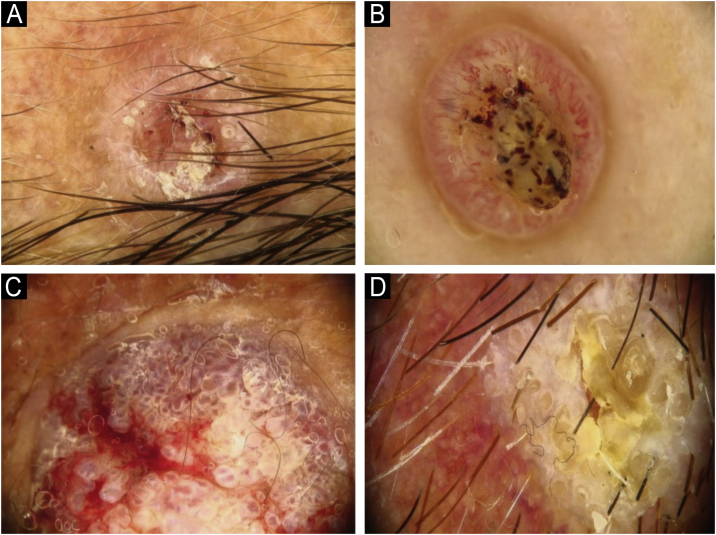
Features of keratinization dermoscopically in cutaneous squamous cell skin cancer: (A) Keratin crust/scale, (B) Central keratin plug, (C) White halos surrounding vessels, (D) White circles surrounding follicles.

**Fig. 2:**
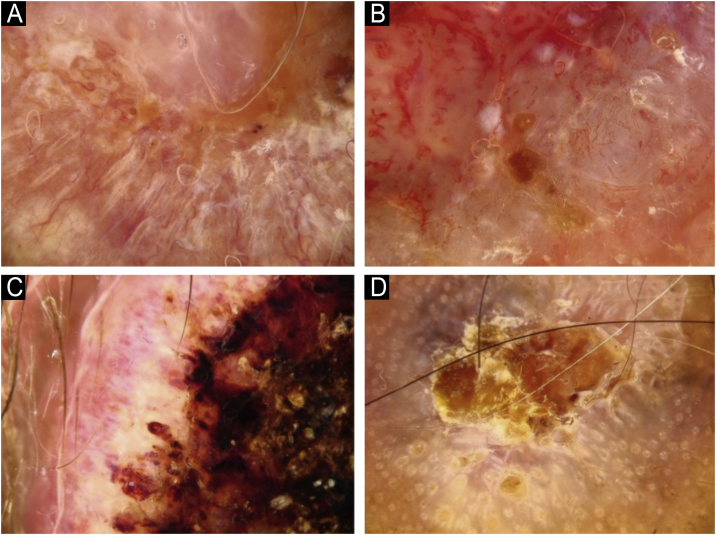
Features of keratinization dermoscopically in cutaneous squamous cell skin cancer: (A) White lines, (B) White clod/dots, (C) White structureless areas, (D) Rosettes.

**Fig. 3:**
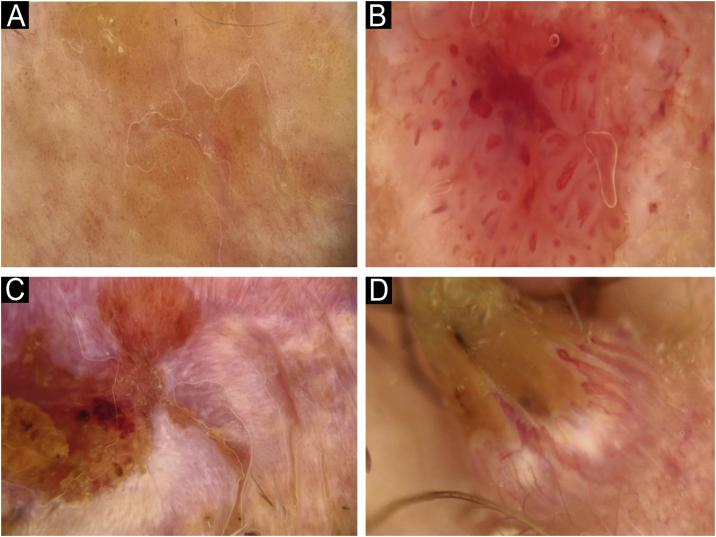
Vascular structures in cutaneous squamous cell carcinoma: (A) Dots, (B) Clods, (C) Linear straight, (D) Linear looped.

**Fig. 4:**
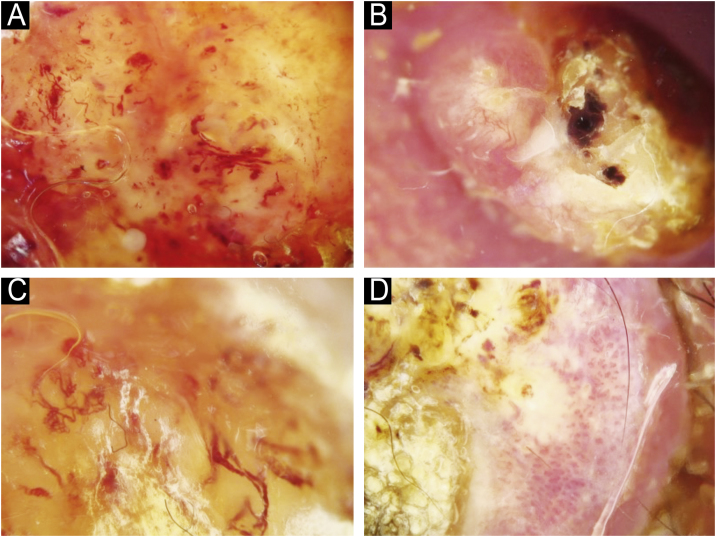
Vascular structures in cutaneous squamous cell carcinoma: (A) Linear curved, (B) Linear serpentine, (C) Linear helical, (D) Linear coiled.

**Fig. 5:**
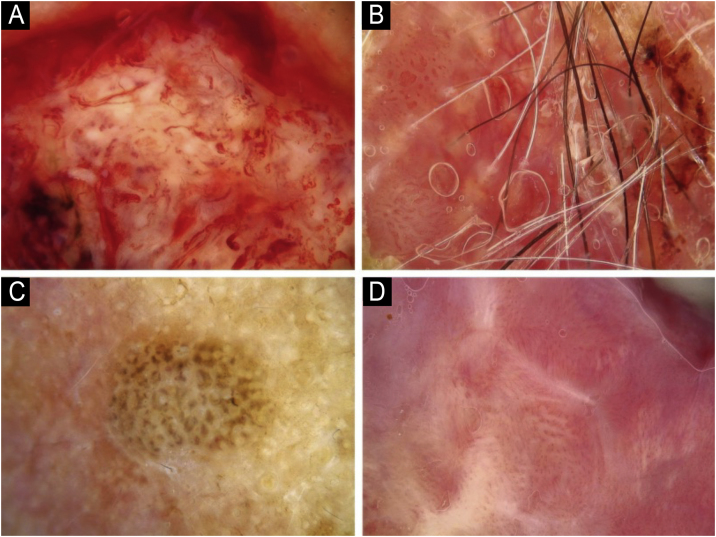
Vascular arrangement in dermoscopy: (A) Random, (B) Clustered, (C) Serpiginous, (D) Linear.

**Fig. 6:**
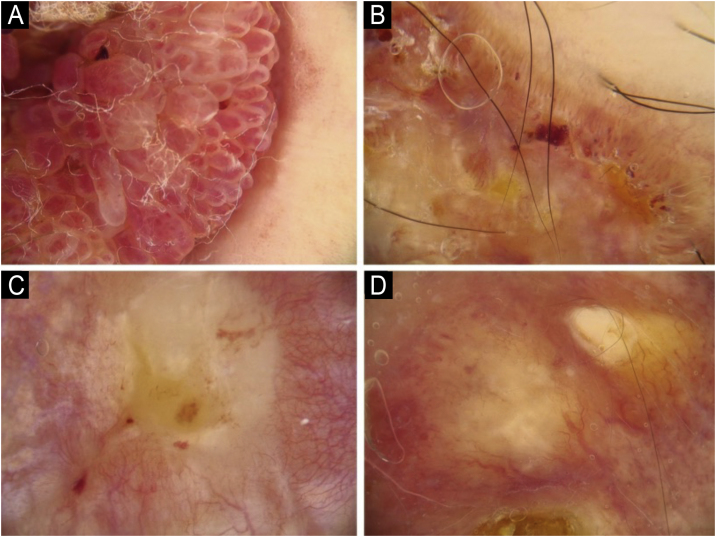
Vascular arrangement in dermoscopy: (A) Centered, (B) Radial, (C) Reticular, (D) Branched.

Using the parameters specified by Sgouros et al. and Rosendahl et al.,^3^, ^8^ the dermoscopic images were evaluated under the following main headings: 1) Features of keratinization (scales, central keratin plug, white halos surrounding vessels, white circles, white clods, white lines, white clods/dots, white structureless areas, and rosettes) (Fig. 1, Fig. 2) 2) Epidermal and dermal losses (ulceration/bleeding, erosion, and blood spots), and 3) Background color (white, pink, red, or mixed). In addition, vessels were classified in more detail according to 1) Vascular pattern (monomorphous, polymorphous, no clear vascular pattern), 2) Vascular structures (dots, clods, linear) (Fig. 3, Fig. 4), and 3) Vascular arrangement (random, clustered, serpiginous, linear, centered, radial, reticular, branched) (Fig. 5, Fig. 6). Linear vascular structures were evaluated as straight, looped, curved, serpentine, helical, or coiled (Fig. 3, Fig. 4).^14^ The dermoscopic terms used are based on the study by Kittler et al.^15^

### Statistical analysis

The data obtained from the study were analyzed using the IBM SPSS Statistics Version 21 package program. Frequency, percentage, mean, and standard deviation were used as descriptive statistical methods in the data analysis. The concordance of the dermoscopic findings identified by the two physicians was evaluated using Cohen’s Kappa statistic. The dermoscopic findings showed high intra-rater agreement and were compared with histopathological findings using chi-square and Fisher exact tests. A p-value below 0.05 was considered statistically significant.

## Results

### Demographic and clinical characteristics of the patients

The mean age of the patients was 69.05 ± 11.92 years, and the male-to-female ratio was 2:1. Most of the lesions were located in the head and neck region (n = 102, 86.4%). Multiple actinic keratosis lesions were detected in 38.1% (n = 45) of the patients at the time of diagnosis. The histopathological classification was well-differentiated cSCC in 50 patients (42.2%), moderately differentiated cSCC in 19 patients (16.1%), poorly differentiated cSCC in 16 patients (13.6%), BD in 10 patients (8.5%), and KA in 23 patients (19.5%) (Table 1).

**Table 1:** Demographic and clinical characteristics and histopathologic diagnoses of patients with cutaneous squamous cell carcinoma.

	n = 118
Age (years), mean ± SD (range)	69.05 ± 11.92 (42–90)
Gender, n (%)	
Male	79 (66.9)
Female	39 (33.1)
Lesion duration (months), mean ± SD (range)	14.78 ± 29.53 (1–240)
Past history of SCC, n (%)	21 (17.8)
Multiple actinic keratosis^a^, n (%)	45 (38.1)
Fitzpatrick phototype, n (%)	
II	49 (41.5)
III	56 (47.5)
IV	13 (11.0)
Location, n (%)	
Head and neck	102 (86.4)
Upper limbs	10 (8.5)
Trunk	4 (3.4)
Lower limbs	2 (1.7)
Lesion site, n (%)^b^	
Sun-exposed	106 (89.8)
Sun-protected	12 (10.2)
Histopathological subtype, n (%)	
Well-differentiated cSCC	50 (42.4)
Moderately differentiated cSCC	19 (16.1)
Poorly differentiated cSCC	16 (13.6)
Bowen’s disease	10 (8.5)
Keratoacanthoma	23 (19.4)

cSCC, cutaneous Squamous Cell Carcinoma.

a Patients with multiple actinic keratosis diagnosed clinically and dermoscopically at the time of cutaneous SCC diagnosis.

b According to the study by Morelló-Vicente et al.^12^

When the distribution of morphological features determined by clinical image analysis was examined according to histopathologic subtype, the authors observed that well-differentiated cSCC most commonly presented with hyperkeratotic papules/plaques (n = 29, 58%), poorly differentiated cSCC with ulcerated papules/plaques and nodules (n = 6, 37.5%), BD with erythematous papules/plaques (n = 8, 80%), and KA with hyperkeratotic nodules (n = 16, 69.5%) (See Supplementary Table S1).

### Dermoscopic features

There was complete agreement between the two physicians’ evaluations for the parameters of dermoscopic pigmentation, absent vessels, ulceration/bleeding, erosion, blood spots, and pink background color (κ = 1.000; p < 0.001). There was also high agreement for all other dermoscopic features (p < 0.001 for all parameters except linear vascular arrangement, p = 0.003) (See Supplementary Table S2).

The most common dermoscopic findings in cSCC included keratin crust/scale (78.9%), linear serpentine vascular structures (74.5%), random vascular arrangement (67.7%), white clod/dots (66.1%), white structureless areas (65.2%), and blood spots (63.5%) (Supplementary Table S3). The distribution of dermoscopic findings in invasive cSCC, BD, and KA is shown in Table 2. Dermoscopic pigmentation was more common in BD patients than in invasive cSCC and KA patients (p = 0.028). Linear serpentine vascular structures were detected more frequently in invasive cSCC and BD than in KA (p = 0.028). The linear coiled vascular arrangement was most common in BD (p = 0.032). Clustered and serpiginous vascular arrangements were also detected more frequently in BD than in invasive cSCC and KA (p = 0.042 and p = 0.006, respectively). Central keratin plug was detected more in KA than in invasive cSCC and BD (p < 0.001). White structureless areas were less common in BD patients than in invasive cSCC and KA patients (p = 0.039). The dermoscopic finding of ulceration/bleeding was most common in invasive cSCC but was not observed in any patients with BD (p < 0.001).

**Table 2:** The distribution of dermoscopic findings in invasive cutaneous squamous cell carcinoma (cSCC), Bowen’s disease, and keratoacanthoma.

Dermoscopic Findings	Invasive cSCC (n = 85)	Bowen’s disease (n = 10)	Keratoacanthoma (n = 23)	Fisher^a^	p-value^a^
	n (%)	n (%)	n (%)	n (%)	n (%)
Dermoscopic pigmentation	4 (4.8)	3 (30.0)	1 (4.3)	6.486	**0.028**
Vascular pattern					
Absent	12 (14.1)	1 (10.0)	8 (34.7)	2.232	0.345
Monomorphous	31 (36.5)	5 (50.0)	7 (30.5)	1.418	0.481
Polymorphous	42 (49.4)	4 (40.0)	8 (34.8)	1.693	0.416
Vascular structures					
Dots					
Clods	46 (54.1)	8 (80.0)	12 (52.2)	2.408	0.293
Linear straight	23 (27.1)	3 (30.0)	5 (21.7)	0.421	0.885
Linear looped	30 (35.3)	2 (20.0)	5 (21.7)	1.959	0.409
Linear curved	51 (60.0)	5 (50.0)	8 (34.8)	4.699	0.097
Linear serpentine	30 (35.3)	1 (10.0)	4 (17.4)	4.437	0.119
Linear helical	68 (80.0)	8 (80.0)	12 (52.2)	6.929	**0.028**
Linear coiled	34 (40.0)	7 (70.0)	5 (21.7)	6.791	**0.032**
Vascular arrangement					
Random	58 (68.2)	7 (70.0)	15 (65.2)	0.180	0.946
Clustered	15 (16.5)	4 (40.0)	1 (4.3)	6.049	**0.042**
Serpiginous	5 (5.9)	4 (40.0)	2 (8.7)	8.772	**0.006**
Linear	9 (10.6)	- (0.0)	2 (8.7)	0.467	0.891
Centered	4 (4.7)	1 (10.0)	1(10.0)	1.649	0.509
Radial	32 (37.6)	1 (10.0)	4 (17.4)	3.033	0.221
Reticular	6 (7.1)	- (0.0)	- (0.0)	1.412	0.486
Branched	29 (34.1)	- (0.0)	12 (52.1)	5.425	0.065
Features of keratinization					
Keratin crust/scale	65 (76.4)	7 (70.0)	21 (91.4)	0.851	0.676
Central keratin plug	29 (34.1)	1 (10.0)	18 (78.5)	18.557	**<0.001**
White halos surrounding vessels	24 (28.2)	3 (30.0)	6 (26.1)	0.163	1.000
White circles surrounding follicles	27 (31.8)	2 (20.0)	5 (21.7)	1.271	0.153
White lines	36 (42.4)	2 (20.0)	8 (34.8)	1.995	0.423
White clod/dots	60 (70.6)	5 (50.0)	13 (56.5)	2.984	0.246
White structureless areas	60 (70.6)	3 (30.0)	14 (60.9)	6.404	**0.039**
Rosettes	19 (22.4)	- (0.0)	3 (13.0)	2.600	0.294
Ulceration/bleeding	34 (40.0)	- (0.0)	2 (8.7)	13.801	**<0.001**
Erosion	24 (28.2)	3 (30.0)	2 (8.7)	3.519	0.165
Blood spots	58 (68.2)	4 (40.0)	13 (56.5)	3.239	0.205
Background color					
White	20 (23.6)	2 (20.0)	12 (47.8)	4.780	0.096
Pink	10 (11.7)	2 (20.0)	2 (8.7)	1.098	0.648
Red	10 (11.7)	2 (20.0)	1 (4.3)	1.993	0.317
Mixed	45 (53.0)	4 (40.0)	8 (34.8)	1.744	0.444

a Fisher’s exact test and Pearson’s Chi-Square test. Statistically significant results (p < 0.05) shown in bold.

The comparison of dermoscopic findings according to invasive cSCC histopathologic differentiation is shown in Table 3. Central keratin plug was more common in patients with well-differentiated cSCC than in patients with moderately and poorly differentiated cSCC (p = 0.021). White circles surrounding follicles, ulceration/bleeding, and red background color were significantly more common dermoscopic findings in poorly differentiated cSCC than other histopathological subtypes of invasive cSCC (p < 0.001, p = 0.001, and p = 0.004, respectively).

**Table 3:** The distribution of dermoscopic findings in invasive cutaneous squamous cell carcinoma according to histopathologic differentiation.

Dermoscopic Findings	Well differentiated (n = 50)	Moderately differentiated (n = 19)	Poorly differentiated (n = 16)	Fisher^a^	p-value^a^
	n (%)	n (%)	n (%)	n (%)	n (%)
Dermoscopic pigmentation	2 (4.0)	1 (5.3)	1 (6.2)	4.250	0.111
Vascular pattern					
Absent	8 (4.0)	3 (15.8)	1 (6.2)	0.309	0.947
Monomorphous	20 (40.0)	5 (26.3)	4 (25.0)	1.293	0.534
Polymorphous	22 (44.0)	11 (57.9)	11 (68.8)	2.710	0.582
Vascular structures					
Dots	25 (50.0)	11 (57.9)	10 (62.5)	0.905	0.630
Clods	9 (18.0)	7 (36.8)	7 (43.8)	5.342	0.058
Linear straight	13 (26.0)	10 (52.6)	7 (43.8)	4.906	0.079
Linear looped	29 (58.0)	11 (57.9)	11 (68.8)	0.633	0.786
Linear curved	14 (28.0)	9 (47.4)	7 (43.8)	2.879	0.250
Linear serpentine	37 (74.0)	16 (84.2)	15 (93.8)	2.925	0.247
Linear helical	8 (16.0)	4 (21.1)	4 (25.0)	0.963	0.688
Linear coiled	16 (32.0)	16 (47.4)	9 (56.3)	3.537	0.181
Vascular arrangement					
Random	31 (62.0)	15 (78.9)	12 (75.0)	2.241	0.324
Clustered	5 (10.0)	6 (31.6)	3 (18.8)	4.647	0.086
Serpiginous	4 (8.0)	- (0.0)	1 (6.3)	1.295	0.698
Linear	4 (8.0)	2 (10.5)	3 (18.8)	1.706	0.427
Centered	2 (4.0)	1 (5.3)	1 (6.3)	0.733	1.000
Radial	22 (44.0)	5 (26.3)	5 (31.3)	2.071	0.352
Reticular	3 (6.0)	2 (10.5)	1 (6.3)	0.783	0.840
Branched	15 (30.0)	9 (47.4)	5 (31.3)	1.921	0.437
Features of keratinization					
Keratin crust/scale	42 (84.0)	13 (68.5)	10 (62.5)	1.749	0.449
Central keratin plug	23 (46.0)	4 (21.1)	2 (12.5)	7.709	**0.021**
White halos surrounding vessels	14 (28.0)	6 (31.6)	4 (25.0)	0.252	0.893
White circles surrounding follicles	9 (18.0)	6 (31.6)	12 (75.0)	18.170	**<0.001**
White lines	25 (50.0)	7 (36.8)	4 (25.0)	3.301	0.187
White clod/dots	35 (68.0)	15 (78.9)	10 (62.5)	2.373	0.294
White structureless areas	39 (78.0)	11 (57.9)	10 (62.5)	3.422	0.193
Rosettes	14 (28.0)	3 (15.8)	2 (12.5)	1.968	0.365
Ulceration/bleeding	15 (30.0)	6 (31.6)	13 (81.2)	13.587	**0.001**
Erosion	14 (28.0)	8 (42.1)	2 (12.5)	3.642	0.173
Blood spots	32 (64.0)	14 (73.7)	12 (75.0)	0.917	0.685
Background color					
White	14 (28.0)	5 (26.3)	1 (6.25)	1.505	0.535
Pink	6 (12.0)	3 (15.8)	1 (6.25)	0.752	0.808
Red	3 (6.0)	1 (5.3)	6 (37.5)	9.580	**0.004**
Mixed	27 (54.0)	10 (52.6)	8 (50.0)	0.142	1.000

a Fisher’s exact test and Pearson’s Chi-Square test. Statistically significant results (p < 0.05) shown in bold.

Based on macroscopic images and clinical features, 20.3% of the lesions were flat. When dermoscopic features were compared between flat and elevated cSCC lesions, the authors found that dermoscopic pigmentation and ulceration/bleeding were prominent in flat lesions (p < 0.001, p = 0.004, respectively), while central keratin plug was more common in elevated lesions (p < 0.001) (Supplementary Table S4).

The dermoscopic characteristics of 14 invasive cSCC and 16 KA patients with nodular morphology are compared in Table 4. The most common dermoscopic findings in nodular lesions were scales (n = 21, 70%), central keratin plug (n = 20, 66.7%), and white structureless areas (n = 18, 60%). Central keratins plug and branched vascular arrangement were observed in 87.5% and 75% of patients with KA, which was statistically more frequent than in invasive cSCC (p ≤ 0.001, p = 0.040, respectively). Dermoscopic findings of white halos surrounding the vessels and clustered vessel arrangement were statistically more common in invasive nodular cSCC compared to nodular KA (p = 0.004 and p = 0.037, respectively).

**Table 4:** Distribution of dermoscopic findings in patients with nodular invasive squamous cell carcinoma and nodular keratoacanthoma.

Dermoscopic Findings	Nodular invasive cSCC (n = 14)	Nodular KA (n = 16)	Total	p-value^a^
	n (%)	n (%)	n (%)	
Dermoscopic pigmentation	- (0.0)	- (0.0)	- (0.0)	‒
Vascular pattern				
Absent	3 (21.4)	3 (18.8)	6 (20.0)	0.919
Monomorphous	5 (35.7)	7 (43.7)	12 (40.0)	0.709
Polymorphous	6 (42.9)	6 (37.5)	12 (40.0)	0.765
Vascular structures				
Dots	7 (50.0)	8 (50.0)	15 (50.0)	1.000
Clods	3 (21.4)	2 (12.5)	5 (16.7)	0.642
Linear straight	3 (21.4)	3 (18.8)	6 (20.0)	1.000
Linear looped	8 (58.1)	7 (43.8)	15 (50.0)	0.464
Linear curved	4 (28.6)	3 (18.8)	7 (23.3)	0.675
Linear serpentine	9 (64.3)	9 (56.2)	18 (60.0)	0.654
Linear helical	3 (21.4)	1 (6.2)	4 (13.3)	0.315
Linear coiled	5 (35.7)	4 (25.0)	9 (30.0)	0.694
Vascular arrangement				
Random	8 (57.1)	8 (50.0)	16 (53.3)	0.696
Clustered	4 (28.6)	- (0.0)	4 (13.3)	**0.037**
Serpiginous	- (0.0)	- (0.0)	- (-)	‒
Linear	- (0.0)	- (0.0)	- (-)	‒
Centered	3 (21.4)	1 (6.2)	4 (13.3)	1.000
Radial	6 (42.9)	5 (31.2)	11 (36.7)	0.510
Reticular	2 (14.3)	- (0.0)	2 (6.7)	0.209
Branched	5 (35.7)	12 (75)	17 (56.7)	**0.040**
Features of keratinization				
Keratin crust/scale	10 (71.4)	11 (68.8)	21 (70.0)	1.000
Central keratin plug	6 (42.8)	14 (87.5)	20 (66.7)	**<0.001**
White halos surrounding vessels	10 (71.4)	3 (18.8)	13 (43.3)	**0.004**
White circles surrounding follicles	7 (50.0)	4 (25.0)	11 (36.7)	0.156
White lines	5 (35.7)	4 (25.0)	9 (30.0)	0.694
White clod/dots	9 (64.3)	7 (43.8)	16 (53.3)	0.261
White structureless areas	9 (64.3)	9 (56.3)	18 (60.0)	0.654
Rosettes	6 (42.8)	3 (18.8)	9 (30.0)	0.236
Ulceration/bleeding	- (0.0)	- (0.0)	- (0.0)	‒
Erosion	1 (7.1)	1 (6.3)	2 (6.7)	1.000
Blood spots	6 (42.9)	7 (43.8)	13 (43.3)	0.961
Background color				
White	7 (50.0)	9 (56.2)	16 (53.3)	0.696
Pink	2 (14.3)	1 (6.2)	3 (10.0)	0.586
Red	1 (7.1)	1 (6.2)	2 (6.7)	1.000
Mixed	4 (28.6)	5 (31.2)	9 (30.0)	1.000

cSCC, Cutaneous Squamous Cell Carcinoma; KA, Keratoacanthoma.

a Fisher’s exact test and Pearson’s Chi-Square test. Statistically significant results (p < 0.05) shown in bold.

## Discussion

The sociodemographic and clinical characteristics of these patients were consistent with previous studies indicating that most cSCC patients are older and male, and lesions are most commonly located in the head and neck region.^16^, ^17^ The incidence of actinic keratosis increases with age, and this increase is higher in the 70-year age range.^18^ Multiple actinic keratosis is one of the risk factors for the development of invasive cSCC.^19^ In a study, the number of multiple actinic keratoses accompanying invasive cSCC was reported as 11.5%.^17^ This rate was found to be higher in the present study. This may be related to the average age in this study being 69.05 and patients being regularly followed up for actinic keratosis and cSCC.

In different studies, dermoscopic pigmentation in cSCCs is reported to vary between 0.01% and 25%.^20^ Its prevalence was found to be higher (5.5%) in pigmented BD.^21^ In the present study, this rate was found to be 6.7% and it was most commonly observed in BD. When evaluating dermoscopic findings, it should be kept in mind that pigmentation may rarely be seen in KA and invasive cSCC.

Among the dermoscopic findings of cSCC, the most common is linear irregular vessels.^3^, ^16^ If linear vessels have more than one turn, it is referred to as a serpentine vessel.^14^, ^15^ The linear irregular vessel structure is also known as a serpentine vessel.^22^, ^23^ In a study by Lallas et al., the dermoscopic findings of invasive cSCC included radial linear irregular and dotted vessels.^16^ Although the studies use different nomenclatures of dermoscopic terms, the dermoscopic features of serpentine and dotted vessels should be considered in the diagnosis of cSCC, consistent with the literature.

Rosendahl et al. determined that the vessel arrangement in KA and invasive cSCC was most frequently random, i.e., nonspecific.^8^ In the present study, a similar distribution was observed in subtypes other than BD. Several studies have demonstrated glomerular vessels and a clustered vascular arrangement to be important findings in the dermoscopic diagnosis of BD.^3^, ^9^ As a result, glomerular vessels and clustered vascular arrangement help to differentiate from KA and invasive cSCC when diagnosing BD.

Well-differentiated invasive cSCCs show an exophytic growth pattern and are present with white structures accompanied by squam on dermoscopy.^16^, ^23^ Sgouros et al. detected the dermoscopic finding of white perifollicular openings more frequently in well-differentiated invasive cSCC.^3^ Manfredini et al. reported that the dermoscopic finding of white structureless areas was more common in well-differentiated invasive cSCC.^24^ In a study conducted in 2024, it was determined that dermoscopic findings such as centrally distributed keratin, white circles, and whitish perivascular halo were more commonly observed in well-differentiated invasive cSCC compared to non-well differentiated invasive cSCC.^25^

Lallas et al. emphasized that a central distribution of scales or keratin on dermoscopic images was a negative predictor of the diagnosis of poorly differentiated invasive cSCC.^16^ In this study, dermoscopic findings of the central keratin plug, keratin crust/scale, and white structureless areas were seen more frequently in well-differentiated invasive cSCCs.

Poorly differentiated cSCC presents clinically as a flat lesion and dermoscopically as red in color, often with abnormal vascular structures.^16^, ^24^ In a study, it is emphasized that the dermoscopic finding of arborizing vessels is important in the diagnosis of poorly differentiated invasive cSCCs. In this study, invasive cSCCs were examined in two groups: well-differentiated and non-well-differentiated.^25^ Both this study and the study by Sgouros et al.^3^ examined invasive cSCC across three different histopathological subtypes. No difference was found between these 3 different histopathological subtypes in terms of dermoscopic findings of vascular structures. Among the patients with poorly differentiated cSCC in the present study, ulceration was detected clinically in 75%, and ulceration/bleeding and red background color were more common dermoscopically. The authors believe that further research is needed on this subject.

In the literature, the dermoscopic findings of white circles surrounding follicles and white halos surrounding vessels are not used separately but referred to collectively as white circles.^16^, ^22^ However, Papageorgiou et al. examined these two dermoscopic findings separately and emphasized that white circles surrounding follicles supported a diagnosis of poorly differentiated invasive cSCC.^24^ The findings of our study support this. In the same study, white halos surrounding vessels were determined to be an important dermoscopic finding for early invasive cSCC. In the present study, white halos surrounding vessels were most frequently detected in well-differentiated cSCC and were important in differentiating nodular invasive cSCC from KA.

The presence of keratin on dermoscopy has high sensitivity in the differentiation of invasive cSCC and KA from cutaneous basal cell carcinoma, one of the other nonmelanocytic skin cancers.^8^ However, the positive predictive value of this dermoscopic finding decreases to 50% when distinguishing cSCC and KA from BD. In the present study, among the dermoscopic signs of keratinization, white circles and white structureless areas were less common in patients with BD. As in this study and the study by Rosendahl et al., the dermoscopic findings of white circles and white structureless areas are supportive of cSCC and KA when differentiating from BD.^8^

In their study, Sgouros et al. emphasized that dermoscopic erosion was mostly seen in patients with BD, while ulceration was mostly seen in patients with invasive cSCC.^3^ In this study, ulcerations were not seen in any of the patients with BD, whereas erosion was detected more frequently than invasive cSCC and KA. When evaluated together with the literature, these findings stand out as important in the dermoscopic differentiation of BD.

Dermoscopic findings seen in KA include a white background, central keratin plug, and linear irregular or hairpin vessels.^3^, ^6^ The literature emphasizes the similarity of dermoscopic findings in KA and invasive cSCC.^8^, ^26^ The most common dermoscopic finding is keratin crust/scale, observed in 100% of KAs and 90% of invasive cSCC.^23^ The dermoscopic findings of keratin crust/scale and white background were most commonly observed in KA in the present study, consistent with the literature. However, the central keratin plug is seen significantly more in KA than in the other subtypes. As this study supports, the central keratin plug is important in the diagnosis of KA.

The most common morphological presentation of KA is nodular lesions with well-defined margins and central hyperkeratotic plugs.^5^ In the present study, 69.5% of KA patients presented with nodular lesions. Nodular and ulcerated lesions are morphologies seen in invasive cSCC.^3^ Dermoscopic differentiation of KA and invasive cSCC is important in patients presenting with similar morphology. Lin et al. argued that the central keratin plug was important in distinguishing KA from nodular cSCC.^23^ The present findings also support this. In the same study, glomerular vascular structures were reported to be the most common vascular structures in nodular cSCC, while no difference in vascular structures or vascular arrangement was found between cSCC and KA. In the study by Pyne et al.,^27^ the branching vessel structure was found to be more common in KA than in invasive cSCC. No comments have been made about lesion morphologies in this study. In the present study, branched vascular arrangement was seen more frequently in patients with nodular KA. In the dermoscopic diagnosis of nodular KA, the presence of central keratin plug and branched vascular arrangement structures should be considered.

Limitations of this study are its retrospective design, the relatively small number of examined lesions in the subgroups, and evaluation of the clinical morphology of the lesions based solely on clinical images, without palpation.

## Conclusions

In this study, specific dermoscopic findings were obtained for the differential diagnosis of BD and nodular KA. However, specific dermoscopic findings were not detected for moderately differentiated invasive cSCC.

The accuracy of the proposed dermoscopic criteria cannot be generalized as a result of the present study, but it will assist clinicians in this regard due to the limited number of studies in the literature.

## Authors’ contributions

Mustafa Ürün: Approval of the final version of the manuscript; study conception, design, and planning; collection, analysis, and data interpretation; writing; critical literature review and critical review of the manuscript.

Yıldız Gürsel Ürün: Approval of the final version of the manuscript; study conception, design, and planning; data interpretation; critical review of the manuscript.

Ömer Faruk Elmas: Approval of the final version of the manuscript; analysis, and data interpretation; critical literature review.

Nuray Can: Approval of the final version of the manuscript; analysis, and data interpretation; writing; critical literature review.

## Financial support

None declared.

## Conflicts of interest

None declared.
