# Supplementary material for: Evaluation of dermoscopic findings in patients with cutaneous squamous cell carcinoma according to histopathological subtype and lesion morphology
Source: An Bras Dermatol. 2025 Feb 13;100(4):101091. doi: 10.1016/j.abd.2024.09.005 (PMC12346027; doi:10.1016/j.abd.2024.09.005)
Supplement: Supplementary file 1 [file mmc1.docx]

ABD-D-24-00483_Supplementary Material

**Supplementary Table 1** Distribution of lesion morphology according to histopathologic subtype.

|  | **Well-differentiated cSCC (n = 50)** | **Moderately differentiated cSCC (n = 19)** | **Poorly differentiated cSCC (n = 16)** | **Bowen’s disease**  **(n = 10)** | **KA**  **(n = 23)** | **n (%)** |
| --- | --- | --- | --- | --- | --- | --- |
| **Erythematous papule/plaque** | 0 (0.0) | 0 (0.0) | 0 (0.0) | 8 (80) | 0 (0.0) | 8 (6.8) |
| **Hyperkeratotic papule/plaque** | 29 (58) | 5 (26.3) | 0 (0.0) | 2 (20) | 6 (26.0) | 42 (35.6) |
| **Hyperkeratotic nodule** | 12 (24.0) | 2 (10.5) | 0 (0.0) | 0 (0.0) | 16 (69.5) | 30 (25.4) |
| **Tumor** | 0 (0.0) | 4 (21.1) | 4 (25) | 0 (0.0) | 0 (0.0) | 8 (6.8) |
| **Ulcerated papule/plaque** | 4 (8) | 5 (26.3) | 6 (37.5) | 0 (0.0) | 0 (0.0) | 15 (12.7) |
| **Ulcerated nodule** | 5 (10) | 3 (15.8) | 6 (37.5) | 0 (0.0) | 1 (4.3) | 15 (12.7) |

cSCC, Cutaneous Squamous Cell Carcinoma; KA, Keratoacanthoma.

**Supplementary Table 2** Concordance of the dermoscopic findings identified by two experts in the diagnosis of cutaneous squamous cell carcinoma.

|  | **Evaluator 2** | **Evaluator 1** | | | | | |
| --- | --- | --- | --- | --- | --- | --- | --- |
|  |  | **Absent** | | **Present** | | **κ^a^** | **p-value^a^** |
|  |  | **n** | **%** | **n** | **%** |  |  |
| **Dermoscopic pigmentation** | Absent | 110 | 100.0 | 0 | 0.0 | 1.000 | **<0.001** |
|  | Present | 0 | 0.0 | 8 | 100.0 |  |  |
| **Vascular pattern** |  |  |  |  |  |  |  |
| Absent | Absent | 97 | 100.0 | 0 | 0.0 | 1.000 | **<0.001** |
|  | Present | 0 | 0.0 | 21 | 100.0 |  |  |
| Monomorphous | Absent | 75 | 100.0 | 0 | 0.0 | 0.963 | **<0.001** |
|  | Present | 2 | 4.7 | 41 | 95.3 |  |  |
| Polymorphous | Absent | 64 | 100.0 | 0 | 0.0 | 0.966 | **<0.001** |
|  | Present | 0 | 0.0 | 54 | 100.0 |  |  |
| **Vascular structures** |  |  |  |  |  |  |  |
| Dots | Absent | 37 | 71.2 | 15 | 24.6 | 0.645 | **<0.001** |
|  | Present | 15 | 22.7 | 51 | 77.3 |  |  |
| Clods | Absent | 80 | 92.0 | 7 | 8.0 | 0.694 | **<0.001** |
|  | Present | 7 | 22.6 | 24 | 77.4 |  |  |
| Linear straight | Absent | 79 | 97.5 | 2 | 2.5 | 0.859 | **<0.001** |
|  | Present | 5 | 13.5 | 32 | 86.5 |  |  |
| Linear looped | Absent | 50 | 93.0 | 4 | 7.0 | 0.897 | **<0.001** |
|  | Present | 0 | 0.0 | 64 | 100.0 |  |  |
| Linear curved | Absent | 80 | 96.4 | 3 | 3.6 | 0.857 | **<0.001** |
|  | Present | 4 | 11.4 | 31 | 88.6 |  |  |
| Linear serpentine | Absent | 29 | 96.7 | 1 | 3.3 | 0.852 | **<0.001** |
|  | Present | 6 | 6.8 | 82 | 93.2 |  |  |
| Linear helical | Absent | 96 | 96.0 | 4 | 4.0 | 0.847 | **<0.001** |
|  | Present | 1 | 5.6 | 17 | 94.4 |  |  |
| Linear coiled | Absent | 70 | 97.2 | 2 | 2.8 | 0.892 | **<0.001** |
|  | Present | 4 | 8.7 | 42 | 91.3 |  |  |
| **Vascular arrangement** |  |  |  |  |  |  |  |
| Random | Absent | 33 | 86.8 | 5 | 6.2 | 0.831 | **<0.001** |
|  | Present | 5 | 13.2 | 75 | 93.8 |  |  |
| Clustered | Absent | 94 | 95.9 | 4 | 4.1 | 0.863 | **<0.001** |
|  | Present | 5 | 25.0 | 15 | 75.0 |  |  |
| Serpiginous | Absent | 105 | 99.0 | 2 | 16.7 | 0.856 | **<0.001** |
|  | Present | 1 | 1.0 | 10 | 83.3 |  |  |
| Linear | Absent | 100 | 93.4 | 7 | 6.6 | 0.278 | **0.003** |
|  | Present | 6 | 54.5 | 5 | 45.5 |  |  |
| Centered | Absent | 110 | 98.2 | 2 | 1.8 | 0.644 | **<0.001** |
|  | Present | 3 | 50.0 | 3 | 50.0 |  |  |
| Radial | Absent | 77 | 95.1 | 4 | 4.9 | 0.904 | **<0.001** |
|  | Present | 1 | 2.7 | 36 | 97.3 |  |  |
| Reticular | Absent | 108 | 99.1 | 1 | 0.9 | 0.880 | **<0.001** |
|  | Present | 1 | 11.1 | 8 | 88.9 |  |  |
| Branched | Absent | 78 | 96.3 | 3 | 3.7 | 0.804 | **<0.001** |
|  | Present | 5 | 14.3 | 30 | 85.7 |  |  |
| **Features of keratinization** |  |  |  |  |  |  |  |
| Keratin crust/scale | Absent | 20 | 66.2 | 5 | 33.8 | 0.890 | **<0.001** |
|  | Present | 8 | 6.7 | 85 | 93.3 |  |  |
| Central keratin plug | Absent | 70 | 95.6 | 0 | 4.1 | 0.947 | **<0.001** |
|  | Present | 3 | 6.0 | 45 | 94.0 |  |  |
| White halos surrounding vessels | Absent | 82 | 96.5 | 3 | 3.5 | 0.779 | **<0.001** |
|  | Present | 3 | 9.0 | 30 | 91.0 |  |  |
| White circles surrounding follicles | Absent | 81 | 98.7 | 3 | 1.3 | 0.807 | **<0.001** |
|  | Present | 9 | 21.4 | 25 | 78.6 |  |  |
| White lines | Absent | 67 | 93.1 | 5 | 6.9 | 0.856 | **<0.001** |
|  | Present | 5 | 10.9 | 41 | 89.1 |  |  |
| White clod/dots | Absent | 33 | 82.5 | 7 | 17.5 | 0.835 | **<0.001** |
|  | Present | 7 | 9.0 | 71 | 91.0 |  |  |
| White structureless areas | Absent | 39 | 95.1 | 2 | 4.9 | 0.944 | **<0.001** |
|  | Present | 1 | 1.3 | 76 | 98.7 |  |  |
| Rosettes | Absent | 88 | 91.7 | 8 | 8.3 | 1.000 | **<0.001** |
|  | Present | 16 | 72.8 | 6 | 27.2 |  |  |
| Ulceration/bleeding | Absent | 82 | 100.0 | 0 | 0.00 | 1.000 | **<0.001** |
|  | Present | 0 | 0.0 | 36 | 100.0 |  |  |
| Erosion | Absent | 89 | 100.0 | 0 | 0.00 | 1.000 | **<0.001** |
|  | Present | 0 | 0.0 | 29 | 100.0 |  |  |
| Blood spots | Absent | 43 | 100.0 | 0 | 0.0 | 1.000 | **<0.001** |
|  | Present | 0 | 0.0 | 75 | 100.0 |  |  |
| **Background color** |  |  |  |  |  |  |  |
| White | Absent | 84 | 100.0 | 0 | 0.0 | 0.979 | **<0.001** |
|  | Present | 0 | 0.0 | 34 | 100.0 |  |  |
| Pink | Absent | 104 | 100.0 | 0 | 0.0 | 1.000 | **<0.001** |
|  | Present | 0 | 0.0 | 14 | 100.0 |  |  |
| Red | Absent | 105 | 100.0 | 0 | 0.0 | 0.958 | **<0.001** |
|  | Present | 0 | 0.0 | 13 | 100.0 |  |  |
| Mixed | Absent | 58 | 95.0 | 3 | 5.0 | 0.949 | **<0.001** |
|  | Present | 0 | 0.0 | 57 | 100.0 |  |  |

^a^ Cohen’s kappa coefficient. Statistically significant results (p<0.05) shown in bold.

**Supplementary Table 3** Dermoscopic findings of all patients with cutaneous squamous cell carcinoma (n = 118).

| **Dermoscopic Findings** | **n (%)** |
| --- | --- |
| **Dermoscopic pigmentation** | 8 (6.7) |
| **Vascular pattern** |  |
| Absent | 21 (17.8) |
| Monomorphous | 43 (36.5) |
| Polymorphous | 54 (45.7) |
| **Vascular structures** |  |
| Dots | 66 (56) |
| Clods | 31 (26.2) |
| Linear straight | 37 (31.3) |
| Linear looped | 64 (54.2) |
| Linear curved | 35 (29.6) |
| Linear serpentine | 88 (74.5) |
| Linear helical | 18 (15.2) |
| Linear coiled | 46 (40) |
| **Vascular arrangement** |  |
| Random | 80 (67.7) |
| Clustered | 20 (16.9) |
| Serpiginous | 11 (9.3) |
| Linear | 11 (9.3) |
| Centered | 6 (5.1) |
| Radial | 37 (31.3) |
| Reticular | 6 (5.0) |
| Branched | 41 (34.7) |
| **Features of keratinization** |  |
| Keratin crust/scale | 93 (78.9) |
| Central keratin plug | 48 (40.6) |
| White halos surrounding vessels | 33 (27.9) |
| White circles surrounding follicles | 34 (28.8) |
| White lines | 46 (38.9) |
| White clod/dots | 78 (66.1) |
| White structureless areas | 77 (65.2) |
| Rosettes | 22 (17.8) |
| Ulceration/bleeding | 36 (30.5) |
| Erosion | 29 (24.5) |
| Blood spots | 75 (63.5) |
| **Background color** |  |
| White | 34 (28.8) |
| Pink | 14 (11.9) |
| Red | 13 (11.0) |
| Mixed | 57 (48.3) |

**Supplementary Table 4** The distribution of dermoscopic findings of cutaneous squamous cell carcinoma according to morphological characteristics.

|  | **Flat cSCC (n=24)** | **Elevated Cscc (n=94)** | **p-value^a^** |
| --- | --- | --- | --- |
| **Dermoscopic Findings** | **n (%)** | **n (%)** |  |
| **Dermoscopic pigmentation** | 8 (33.3) | - (0.0) | <0.001 |
| **Vascular pattern** |  |  |  |
| Absent | 6 (25.0) | 15 (16.0) | 0.384 |
| Monomorphous | 8 (33.3) | 35 (37.2) | 0.461 |
| Polymorphous | 10 (41.7) | 44 (46.8) | 0.863 |
| **Vascular structures** |  |  |  |
| Dots | 16 (66.6) | 50 (53.1) | 0.858 |
| Clods | 8 (33.3) | 23 (24.5) | 0.637 |
| Linear straight | 7 (29.1) | 30 (31.9) | 0.575 |
| Linear looped | 16 (66.6) | 48 (51.0) | 0.271 |
| Linear curved | 5 (20.8) | 30 (31.9) | 0.289 |
| Linear serpentine | 18 (75.0) | 70 (74.4) | 0.717 |
| Linear helical | 2 (8.3) | 16 (17.0) | 0.518 |
| Linear coiled | 10 (41.6) | 36 (38.2) | 0.694 |
| **Vascular arrangement** |  |  |  |
| Random | 18 (75.0) | 62 (65.9) | 0.696 |
| Clustered | 6 (25.0) | 14 (14.8) | 0.480 |
| Serpiginous | 4 (10.7) | 7 (7.4) | 0.261 |
| Linear | 1 (4.1) | 10 (10.6) | 0.463 |
| Centered | 2 (8.3) | 4 (4.2) | 1.000 |
| Radial | 5 (20.8) | 32 (34.0) | 0.249 |
| Reticular | 2 (8.3) | 4 (4.2) | 1.000 |
| Branched | 11 (45.8) | 30 (31.9) | 0.223 |
| **Features of keratinization** |  |  |  |
| Keratin crust/scale | 20 (83.3) | 73 (77.6) | 0.584 |
| Central keratin plug | - (0.0) | 48 (51.0) | <0.001 |
| White halos surrounding vessels | 7 (29.2) | 26 (27.6) | 0.721 |
| White circles surrounding follicles | 7 (29.2) | 20 (21.2.2) | 0.520 |
| White lines | 12 (50.0) | 34 (48.9) | 0.180 |
| White clod/dots | 16 (66.6) | 62 (65.9) | 0.690 |
| White structureless areas | 12 (50.0) | 65 (69.1) | 0.079 |
| Rosettes | 4 (16.7) | 15 (16.0) | 0.544 |
| Ulceration/bleeding | 13 (54.2) | 23 (24.5) | 0.007 |
| Erosion | 5 (20.8) | 24 (25.5) | 0.957 |
| Blood spots | 17 (70.8) | 58 (61.7) | 0.357 |
| **Background color** |  |  |  |
| White | 3 (12.5) | 32 (31.9) | 0.059 |
| Pink | 4 (16.7) | 10 (10.6) | 0.480 |
| Red | 3 (12.5) | 10 (10.6) | 0.738 |
| Mixed | 15 (62.5) | 42 (44.6) | 0.349 |

cSCC, Cutaneous Squamous Cell Carcinoma.

^a^ Fisher’s exact test and Pearson’s Chi-Square test. Statistically significant results (p < 0.05) shown in bold.
